# A spatial regression approach to modeling urban land surface temperature

**DOI:** 10.1016/j.mex.2023.102022

**Published:** 2023-01-19

**Authors:** Abdur-Rahman Belel Ismaila, Ibrahim Muhammed, Bashir Adamu

**Affiliations:** a Department of Urban and Regional Planning, Faculty of Environmental Sciences, Modibbo Adama University, Yola, P.M.B. 2076, Yola, Adamawa State, Nigeria; b Department of Surveying and Geoinformatics, Faculty of Environmental Sciences, Modibbo Adama University, Yola, P.M.B. 2076, Yola, Adamawa State, Nigeria; c Department of Geography, Faculty of Environmental Sciences, Modibbo Adama University, Yola, P.M.B. 2076, Yola, Adamawa State, Nigeria

**Keywords:** Spatial modeling, Spatial lag model, Spatial error model, Surface temperature, Urban area

## Abstract

Land surface temperature (LST) is the instantaneous radiative skin temperature of land obtained from satellite sensors. Measured by visible, infrared or microwave sensors, the LST is useful in determining thermal comfort for urban planning. It also serves as a precursor to many underlying impacts such as health, climate change and the likelihood of rainfall. Due to the paucity of observed data because of cloud cover or rain-bearing clouds in the case of microwave sensors, it is necessary to model LST for the purpose of forecasting. Two spatial regression models were employed: the spatial lag model and the spatial error model. Using Landsat 8 and Shuttle Radar Topography Mission (SRTM), these models can be studied and compared in terms of their robustness in reproducing LST. Whereas LST is to be the independent variable, built-up area, water surface, albedo, elevation, and vegetation are to be considered as dependent variables and their relative contributions to LST examined.

• Modeling LST based on spatial regression models with calculated LST as independent variable.
• Dependent variables to be considered are normalised difference Built-up index (NDBI), normalised difference vegetation index (NDVI), modified normalised difference water index (MNDWI), albedo and elevation.
• The models were validated using k-fold cross validation method, mean square error and standard deviation.

## Graphical abstract

**Figure:**
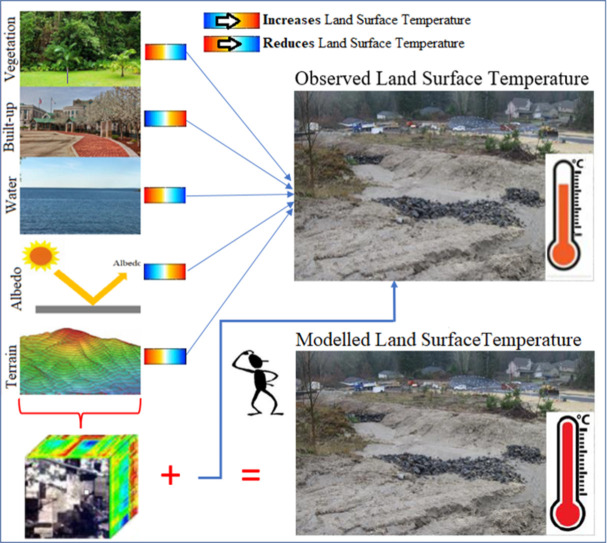

Specifications table

**Table:** 

Subject Area:	Environmental Science
More specific subject area:	Land Surface Temperature Modelling
Method name:	Spatial modeling
Name and reference of original method:	Ismaila A.-R. B., Muhammed, I., Adamu, B., 2022. modeling land surface temperature in urban areas using spatial regression models, Urban Clim. 44(101,213): 1–14. https://doi.org/10.1016/j.uclim.2022.101213
Resource availability:	Earth Explorer webservice from United States Geological Services. Both datasets (Landsat and SRTM) used are obtainable there. The datasets are obtained by choosing an area of study under search criteria, choosing datasets and additional criteria that include a percentage of cloud cover before getting the results. www.earthexplorer.usgs.gov

## Method details

Physical environment and geophysical features are investigated in order to better understand our environment and harness its resources. These studies are done using *in situ*-based measurements using probes. However, there are drawbacks due to inability of probes to sample large areas and be able to have repeated measurements over time. These limitations are handled by satellite measurements. Satellites are being developed to assist us in mapping our environment spatially over large areas using short time repeat cycles.

The Landsat satellite series is useful for environmental research. Landsat data is used in a variety of applications around the world, including agriculture, forestry, and range resource management, land use and mapping, geology, hydrology, and coastal resources. Imagery with a moderate spatial resolution and multispectral capability is well suited to a wide range of applications ranging from modeling of population dynamics of disease vectors in association with habitat features to support for emergency response, environmental comfort, disaster relief and predictions [8]. Other areas include vector-borne diseases mapping such as malaria and dengue fever locations. These are compared to terrain data to study areas that are particularly conducive to their breeding in terms of height and moisture. These, along with rainfall, can be incorporated into vector capability models (VCAP) to simulate disease endemism [6].

Processing these data require prior knowledge of how satellite sensors work as well as understanding of atmospheric attenuation that occurs as sensor signals pass through the atmosphere. However, with the exception of a few issues such as cloud and haze, which are managed by atmospheric correction by end users, the majority of pre-processing work is performed by data providers prior to the release of data to the public.

The choice of data resolution in terms of both spatial and temporal resolutions is necessary to arrive at sensible results. Large areas can tolerate low-resolution data while small areas only require high-resolution data sets. The temporal resolution, however, depends on the kind of analysis such as the interest in studying changes over time. This study covers an area greater than 8000 hectares of land covering both urban and suburban areas. Therefore, the choice of Landsat and SRTM DEM both at 30 m spatial resolutions is an excellent choice.

The objective of the paper was to measure quantitatively the relationship between LST and other variables in a tropical Savannah city, Yola Nigeria using the two data sets. Landsat was used to compute LST, NDBI, NDVI, MNDWI and surface albedo, while SRTM DEM was used to compute elevation [7]. Our regression model considers LST as dependent variable and others as independent variables. This is to be able to understand how each of the independent variables affects the dependent variable. These are:

a) Dependent variable: Land surface temperature (LST).
b) Explanatory variables:
  I. Normalized difference built-up index (NDBI) for built-up areas.
  II. Normalized difference vegetation index (NDVI) for vegetation cover.
  III. Modified normalized difference water index (MNDWI) for water surface.
  IV. Albedo, which is the measure of the diffuse reflection of solar radiation out of the total solar radiation budget.
  V. Digital elevation model (DEM), which is the satellite Shuttle Radar Topography Mission (SRTM) data.

**Fig. 1:**
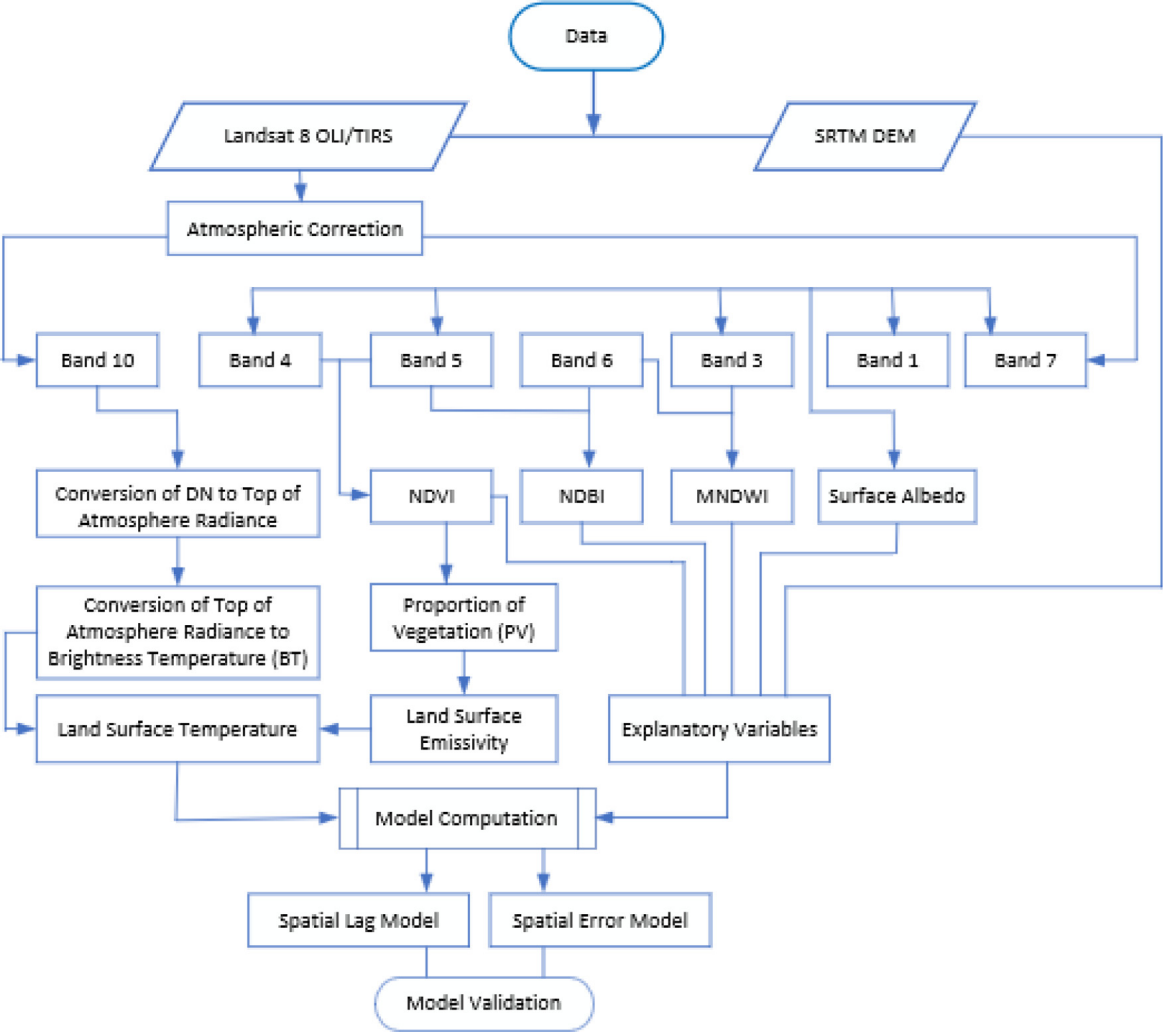
The methodology flowchart.

Data processing and analysis was conducted using graphic user interface (GUI) software packages that include ArcGIS, ENVI and GeoDa. The general methodology is shown in Fig. 1.

### Atmospheric correction

The atmosphere absorbs and scatters various wavelengths of the visible spectrum, which must pass through the atmosphere twice, once from the sun to the object and again as it returns up to the image sensor. To obtain accurate at-sensor radiance, these distortions must be corrected. Hence, atmospheric correction was employed to eliminate the effects of the atmosphere on reflectance values. The technique used is Dark Object Subtraction via ENVI software. This was applied to Landsat data, which is optical sensor data that required correction. It removed the effects of atmospheric scattering from the images by subtracting a pixel value from each band that represents a background signature. This value can be the minimum of the band or an average based on a region of interest (ROI).

### Derivation of land surface temperature

Landsat 8 OLI/TIRS images were used to compute LST, NDBI, NDVI, MNDWI and Albedo. The LST parametisations were extracted from the metadata file downloaded with the Landsat 8 OLI/TIRS data. All conversions were performed according to Landsat 8 parametizations provided by USGS [15]. Other indices such as NDVI is based on Sobrino et al., [13] and Palafox-Juárez et al., [9], NDBI [17], and MNDWI [16].

The LST was processed as follows; a) conversion of band 10 digital numbers to at-sensor spectral radiance, b) conversion of spectral radiance to brightness temperature, c) computation of land surface emissivity (ε), and d) computation of LST using the single window method [9,10] as shown in Eq. (1).

(1) $$L\lambda =ML*Q_{cal}+AL-O_{i}$$

where, ML represents a multiplicative rescaling factor of the specific band, $Q_{cal}$ is the value of the thermal band image, *AL* is the rescaling specific factor of the band, and $O_{i}$ is the correction of the thermal band.

BT = At sensor brightness temperature radiance. This is calculated in degree Celsius as;

(2) $$BT=\left(\frac{K_{2}}{ln\left(\frac{K_{1}}{L\lambda }\right)+1}\right)-273.15$$

where, $K_{1}$ and $K_{2}$ represent the band-specific thermal conversion constants obtainable for Landsat metadata. The BT results will be in degree Celsius. Next is the Normalised Difference Vegetation Index

(3) $$NDVI=\;\frac{\text{NIR}-\text{RED}}{\text{NIR}+\text{RED}}$$

Proportion of vegetation (*PV*) is calculated prior to emissivity ε using a semi empirical method based on NDVI

(4) $$PV={\left\lceil \frac{NDVI\;-\;NDVI_{min}}{NDVI_{max}-NDVI_{min}}\right\rceil }^{2}$$

where,$\;NDVI_{max}=\;0.5\;\text{and}\;NDVI_{min}=\;0.2$,

(5) ε=0.004*PV+0.986

where, 0.004 corresponds to the average emissivity value of bare soil, 0.986 the average emissivity values of the vegetated areas, and PV is the proportion of vegetation [14]. Next is wavelength of radiance ρ for each band.

(6) $$\rho =\frac{hc}{\delta }10^{6}$$

where,

*h* - Plank's constant = 6.62607004 × 10^−34^,
*c* - Speed of light = 2.998×10^8^,
δ- Boltzmann's constant = 1.3807×10^−23^

The multiplication by 10^6^ is to convert values in meters to micrometers.

(7) $$LST=\left[\frac{\text{BT}}{\left(1+\left[\left(\frac{\lambda BT}{\rho }\right)In\;\varepsilon \lambda \right]\right)}\right]$$

where, LST = Land Surface Temperature in degrees Celsius, λ is the center wavelength of emitted radiance for Band 10 (10.8 µm).

### Extraction of explanatory variables

NDBI, NDVI, and MNDWI, on the other hand, were processed as follows: a) conversion of all relevant bands to top of atmosphere (ToA) reflectance.

(8) $$NDBI=\frac{\text{SWIR}1-\;\text{NIR}}{\text{SWIR}1+\;\text{NIR}}$$

(9) $$NDVI=\;\frac{\text{NIR}-\text{RED}}{\text{NIR}+\text{RED}}$$

(10) $$MNDWI=\;\frac{\text{GREEN}-\;\text{SWIR}1}{\text{GREEN}+\;\text{SWIR}1}$$

where, SWIR1 is the short-wave infrared band, NIR is the near-infrared band, RED is the red band, and GREEN is the green band.

Albedo was retrieval from the Landsat 8 OLI data based on Landsat 7 ETM+ equivalent [12]:

(11) $$Albedo=\;\frac{0.356\alpha 2+0.130\alpha 4+0.373\alpha 5+0.085\alpha 6+0.072\alpha 7-0.0018}{0.356+0.130+0.373+0.085+0.072}$$

where, *α* represents Landsat 8 OLI bands 2, 4, 5, 6 and 7.

### Correlation between LST and other remote sensing indices

After extracting the variables, we examined the relationship between LST and the variables. This indicates whether LST is positively or negative correlated with those variables, and whether the correlations are strong, moderate or weak. The correlations are based on time series of pixels from the spatial data sets obtained after vectorization of the matrices. Finally, using these values, respective scatter plots were generated from which the coefficients of correlation and line plots overlaid.

### Diagnosis for spatial autocorrelation in the variables

The presence or absence of spatial autocorrelation in the variables was assessed. Spatial autocorrelation is the correlation among data values, strictly due to the relative location proximity of neighbourhood pixels. We employed the robust Lagrange-multiplier test strategy of Anselin et al. [4], which offered tests of spatial-autoregressive lag or of error against independence that are robust to the presence of the other autoregressive processes.

### Modeling LST using spatial regression

This study employed the spatial lag model (SLM) and spatial error model (SEM). Both models were implemented using GeoDa software.

The SLM takes the form of Eq. (12).

(12) $$LST_{SLM}=\rho WLST_{ob}+X\beta +\varepsilon$$

where, *LST* is a vector of spatially lagged dependent variables, *ρ* the spatial lag factor. *W* is a weight matrix, $LST_{ob}$ is a spatially lagged dependent variable, *X* is a matrix of observations of NDBI, NDVI, MNDWI, albedo, and elevation, *β* is a vector of coefficients for the regression model, and *ε* is a vector of explanatory variables and identically distributed error terms.

The SEM takes the form described by Eq. (13):

(13) $$LST_{SEM}=X\beta +u$$

where, *u* = λWu + ε where, *u* is the error term expressing spatial dependence, λ is the autoregressive coefficient. All other terms are as previously defined.

The model comparison include the following:

i) Log-likelihood: The log-likelihood function was used to derive the maximum likelihood estimator of the parameters. The estimator is obtained by solving, that is, by finding the parameter that maximizes the log-likelihood of the observed sample. This is the same as maximizing the likelihood function. It is usually calculated with software due to complexity, and in this case we used GeoDa regression software.
ii) Akaike information criterion (AIC): is an estimator of prediction error and thereby relative quality of statistical models for a given set of data [1]. Given a collection of models for the data, AIC estimates the quality of each model, relative to each of the other models. Thus, AIC provides a means for model selection. The formula for AIC depends upon the statistical model. Suppose that we have a statistical model of some data. Let k be the number of estimated parameters in the model. Let *L* be the maximized value of the likelihood function for the model. Then the AIC value of the model is the following [5]:
  (14) AIC=2k−2ln(L)
iii) Schwarz criterion (SC): The Schwarz criterion is an index to help quantify and choose the least complex probability model among multiple options. It is also called Bayesian Information Criterion (BIC). This approach compares efficiencies of different models at predicting outcomes. Efficiency is measured by creating an index of each model's parameters using a log-likelihood function.
  (15) SC=log(n)k−2log(L(U))
  where, *n* = sample size, *k* = number of parameters, *U*= set of all parameter values
  L(U)= likelihood of the model returning the data we have, and then tested with maximum likelihood values of U. This procedure is repeated for each model. The model with the highest log likelihood, or with the lowest AIC or SC is the best [3].

### Model performance

Verification for accuracy and performance of the model was done using a number of metrics (Eq. (16) & 17). The models were validated using a *k-*fold cross-validation (CV), [11]. This approach split the dataset into 5 folds, of approximately equal size. The division is an optimal standard for computational efficiency. One can decide to have more than 5 folds. The training dataset constitutes 80% while 20% represent testing set [2], which is a standard model prediction criteria. The metrics of validation of performance include:

i. The first fold is considered as a validation set, and the remaining *4* folds are treated as training set.
ii. The mean squared error,*MSE*_1_ is then computed on the observations in the held-out fold.
  (16) $$MSE=\frac{1}{n}\sum_{i=1}^{n}{\left(LST_{pred}-LST_{obs}\right)}^{2}$$
iii. This procedure is repeated k times;
  a. Each time, a different group of observations is treated as a validation set.
  b. This process results in k estimates of the test error, *MSE*_1_.....*MSE_k_*.
iv. The *k*-fold CV estimate is computed by averaging these values:
  (17) $$CV_{\left(k\right)}=\frac{1}{k}\sum_{i=1}^{k}MSE_{i}$$
v. Compute standard deviation.

## Declaration of interests

The authors declare that they have no known competing financial interests or personal relationships that could have appeared to influence the work reported in this paper.

## Data Availability

I have shared the link to my data Application of Landsat-8 OLI/TIRS and digital elevation model dataset in spatial modelling of land surface temperature (Original data) (Mendeley Data)
